# *In vitro* and *in silico* study of biological effects on cancer cells in the presence of metallic materials during radiotherapy

**DOI:** 10.1093/jrr/rrae062

**Published:** 2024-08-22

**Authors:** Takuya Nagano, Yusuke Matsuya, Atsushi Kaida, Hitomi Nojima, Takuya Furuta, Kaoru Sato, Ryoichi Yoshimura, Masahiko Miura

**Affiliations:** Department of Radiation Therapeutics and Oncology, Tokyo Medical and Dental University, 1-5-45 Yushima, Bunkyo-ku, Tokyo 113-8519, Japan; Nuclear Science and Engineering Research Center, Japan Atomic Energy Agency, 2-4 Shirakata, Tokai, Ibaraki 319-1195, Japan; Faculty of Health Sciences, Hokkaido University, Kita-12 Nishi-5, Kita-ku, Sapporo, Hokkaido 060-0812, Japan; Department of Dental Radiology and Radiation Oncology, Tokyo Medical and Dental University, 1-5-45 Yushima, Bunkyo-ku, Tokyo 113-8519, Japan; Department of Dental Radiology and Radiation Oncology, Tokyo Medical and Dental University, 1-5-45 Yushima, Bunkyo-ku, Tokyo 113-8519, Japan; Nuclear Science and Engineering Research Center, Japan Atomic Energy Agency, 2-4 Shirakata, Tokai, Ibaraki 319-1195, Japan; Nuclear Science and Engineering Research Center, Japan Atomic Energy Agency, 2-4 Shirakata, Tokai, Ibaraki 319-1195, Japan; Department of Radiation Therapeutics and Oncology, Tokyo Medical and Dental University, 1-5-45 Yushima, Bunkyo-ku, Tokyo 113-8519, Japan; Department of Dental Radiology and Radiation Oncology, Tokyo Medical and Dental University, 1-5-45 Yushima, Bunkyo-ku, Tokyo 113-8519, Japan

**Keywords:** X-ray therapy, high-z dental metal, dose enhancement, sensitizer enhancement ratio, Monte Carlo simulation, cell experiment

## Abstract

X-ray therapy aims to eliminate tumours while minimizing side effects. Intense mucositis is sometimes induced when irradiating the oral cavity with a dental metal crown (DMC). However, the underlying mechanisms of such inducing radiosensitization by DMC remain uncertain. This study explored the radiosensitizing mechanisms around DMCs in an interdisciplinary approach with cell experiments and Monte Carlo simulation with the PHITS code. Clonogenic survival and nuclear 53BP1 foci of a cell line derived from cervical cancer cells (HeLa cells) were measured post-irradiation with therapeutic X-rays near high-Z materials such as Pb or Au plates, and the experimental sensitizer enhancement ratio (SER) was obtained. Meanwhile, the dose enhancement ratio (DER) and relative biological effectiveness for DNA damage yields were calculated using the PHITS code, by considering the corresponding experimental condition. The experiments show the experimental SER values for cell survival and 53BP1 foci near metals are 1.2–1.4, which agrees well with the calculated DER values. These suggest that the radiosensitizing effects near metal are predominantly attributed to the dose increase. In addition, as a preclinical evaluation, the spatial distributions of DER near DMC are calculated using Computed Tomography Digital Imaging and Communications in Medicine (CT-DICOM) data and a simple tooth model. As a result, the DER values evaluated using the CT-DICOM data were lower than those from a simple tooth model. These findings highlight the challenge of evaluating radiosensitizing effects near DMCs using Digital Imaging and Communications in Medicine (DICOM) images due to volume-averaging effects and emphasize the need for a high-resolution (<1 mm) dose assessment method unaffected by these effects.

## INTRODUCTION

Radiotherapy, a method for eradicating solid tumours, aims to achieve local tumour control while minimizing harm to adjacent organs at risk [1–3]. In external radiotherapy particularly for head and neck cancer (oropharyngeal cancer, tongue cancer, gingival cancer and metastatic tongue cancer), both oral metallic materials like DMCs or implants and solid tumours are often simultaneously irradiated with X-ray beams [1]. During such an irradiation situation, backscattered X-rays potentially increase the energy deposition on the side of the metal opposite to the beam direction, whereas forward scattering reduces the dose on the side of the metal facing the beam [4]. The backscattered X-rays with energy lower than primary X-rays can be incident on tissues near the metal. Some dosimetric studies have reported that X-rays and electrons backscattered at the surface of metals can enhance the absorbed dose by ~1.2-fold at 1 mm upstream of the metal when a 2 mm-thick dental metal crown (DMC) is irradiated [4–7]. From the standpoint of radiation protection, it is crucial for evaluating such maximal biological impacts. In actual clinical settings, the radiation field often includes the oral mucosa when cervical lymph nodes are prophylactically irradiated. Additionally, irradiating in the presence of DMC in the oral cavity can lead to serious side effects, such as severe mucositis [4, 8]. Given the risk of severe side effects when irradiating near DMCs, it is also essential to understand the mechanisms behind the increased radiosensitivity in the surrounding tissues as well as evaluating the maximal impacts from the viewpoint of radiation protection. In recent decades, the biological impacts of gold nanoparticles have been attracted [9]. Clarifying these radiosensitization processes by metal (e.g. gold) will help develop strategies to mitigate adverse effects and optimize treatment outcomes for head and neck cancer patients with DMCs.

From a dosimetry perspective, several reports have detailed both dose measurement methods and Monte Carlo (MC) simulations for assessing dose distribution around DMC [4]. This dosimetric analysis has shown that energy is locally deposited within regions proximal to metals, typically <1–2 mm [6]. Consequently, MC simulation has proven invaluable for evaluating dose enhancement with high spatial resolution. To date, using such simulation techniques, research has been conducted on methods to mitigate localized mucositis during radiation therapy [4, 7]. In addition to the local dose enhancement, the scattered X-rays by metals can potentially enhance the biological impact due to their low energy [10, 11]. Considering these dosimetric and biological aspects, while an enhancement of radiosensitivities in the presence of DMC is anticipated, the radiosensitizing mechanisms near metals remain uncertain because of insufficient experimental data. Typically, the enhancement degree is expressed by using the sensitizer enhancement ratio (SER), which denotes the ratio of the dose required to induce biological effects without a sensitizer to the dose needed with a sensitizer. To precisely understand the biological mechanisms, it is essential to experimentally and theoretically evaluate the SER values for surviving fraction and DNA double-strand break (DSB) (linked intrinsically to cell death) by combining dose assessment with MC simulation.

In this study, to theoretically clarify the radiosensitizing mechanisms, we used a general-purpose MC simulation code for simulating radiation transport in matters, Particle and Heavy Ion Transport code System (PHITS), which allows the estimates of the dose increases and the biological impact (DNA damage estimation [12]) based on track-structure simulation of the scattered X-rays. In parallel, we also performed the cell experiments, i.e. clonogenic survival assay and 53BP1 foci formation assay for measuring the experimental SER values for survival and DSB. Using the interdisciplinary approach including MC simulation and cell experiments, herein, we explored the biological effects on cancer cells located near oral metallic materials following therapeutic X-ray irradiation for clarifying the radiosensitizing mechanisms near a DMC. Finally, we present the spatial distribution of radiosensitizing effects near DMC predicted based on CT-DICOM data and discuss the current issues and future prospects.

## MATERIALS AND METHODS

### Preparation of metal discs

**Fig. 1:**
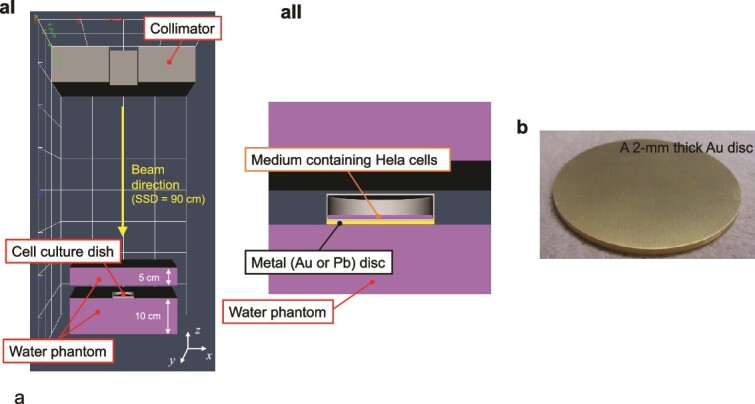
Irradiation setup for a cell culture dish using a linear accelerator. (aI) Cross-sectional view of the simulated collimator, cell culture dish and water phantom in PHITS. (aII) Enlarged cross-sectional view of the simulated dish, culture medium and metal disc in PHITS. It should be noted that the metal disc is precisely machined to fit the bottom of the dish, ensuring no air gap between the dish and the metal disc. (b) A 2 mm-thick Au disc used in the experiment.

In this study, we performed *in vitro* experiments using gold (Au) and lead (Pb) as representative high-Z materials of DMCs. Au is a rare metal, while Pb is known for its excellent machinability. These metal discs were placed under the cell culture dishes (Thermo Scientific, φ60 mm). The Au discs were purchased from Medikit Corporation (Tokyo, Japan) and attached to a plastic base that precisely fit the entire bottom surface of the culture dish, ensuring no air gap between the dish and the metal disc. The thickness of the dish of the base is 1 mm as shown in Fig. 1aI. The Pb discs were cut from a 0.5 mm-thick Pb sheet provided by Hikari Co., Ltd. (Osaka, Japan) and machined to fit the bottom of the culture dish precisely, eliminating any air gap. We also investigated the biological effects in the presence of various shapes and conditions of metal sheets (see Fig. S1 in the supplementary material).

### Cell line and cell culture

Human cervical carcinoma HeLa cells, modified to stably express the Fucci probe (HeLa-Fucci (SA)), were used in this study. The cells were provided by RIKEN BRC through the National Bio-Resource Project of MEXT, Japan. The radiosensitivity of the HeLa cells was evaluated using the clonogenic survival assay, and the experimental SER for survival (SER_SF_) was calculated. Although this study aims to evaluate the biological effects in the body around metal using DMCs as an example, the HeLa cells were chosen for the *in vitro* experiments due to their robust cell proliferation and consistent reproducibility. In addition, the other reason for using HeLa cells instead of normal cells is that their radiosensitivity has been well documented in the literature, e.g. in the Particle Irradiation Data Ensemble (PIDE) database [13].

The HeLa cells were cultured in Dulbecco’s Modified Eagle Medium (DMEM; Sigma-Aldrich, St. Louis, MO, USA) supplemented with 10% foetal bovine serum, 100 U/ml penicillin and 100 μg/ml streptomycin. The DMEM contained a low glucose concentration of 1000 mg/L. Cells were maintained in a humidified incubator at 37°C with 5% CO_2_.

### Irradiation conditions

The HeLa cells were irradiated using Varian Clinac 4EX and Clinac 6EX linear accelerators (Varian Medical Systems, Palo Alto, CA, USA) with therapeutic X-ray energies of 4, 6 and 10 MV at a dose rate of 2.4 Gy/min. The cell culture dish was placed between two layers of solid water: a 10 cm-thick layer beneath the dish and a 5 cm-thick layer above it. Au and Pb disc-shaped metals, cut to the same size as the culture dish, were placed directly under the dish, ensuring no air gap between the metal discs and the dish bottom, to investigate the biological effects of high-Z materials on the irradiated cells. The cell layer was positioned at the isocentre (100 cm source–surface distance) and irradiated with a 20 cm × 20 cm radiation field (see Fig. 1).

### Clonogenic survival assay

The dose–response curve of cell survival obtained from the clonogenic survival assay serves as a quantitative indicator of radiosensitivity. In this study, the radiosensitivity of the HeLa cells was evaluated by varying the X-ray energy (i.e. 4, 6 and 10 MV) and using Au or Pb discs. Cells were seeded in triplicate in 60 mm dishes and irradiated at various X-ray doses. Following irradiation, cells were incubated for 10 days at 37°C with 5% CO_2_. Colonies were then fixed in 4% paraformaldehyde and stained with crystal violet. Colonies consisting of 50 or more cells were counted, and the colony formation efficiency was calculated. Colony formation efficiency was expressed as the average of three independent experiments. Survival curves were fitted using the linear-quadratic model and calculated with Prism 9 (GraphPad Software, La Jolla, CA, USA, 2021).

### Immunofluorescent staining for assessing DSB yield

DSB yield was determined after 2 Gy irradiation, providing insights into the biological effects of metal discs at a clinically relevant dose during radiotherapy. To investigate DNA damage response, changes in the localization of 53BP1, a protein involved in the DNA damage response, were observed by fluorescence microscopy. The HeLa cells were seeded on Lab-Tek Chamber Slides (Nunc, Rochester, NY) and cultured for 24 h at 37°C in a 5% CO_2_ atmosphere. Following X-ray irradiation, the cells were immediately fixed in 4% paraformaldehyde in PBS for 30 min. After fixation, the cells were permeabilized with 0.1% Triton X-100 in PBS (PBS-T) for 10 min, and non-specific binding sites were blocked with 10% normal goat serum (Life Technologies) for 30 min. The cells were then incubated with a rabbit-derived primary monoclonal antibody against 53BP1 (ab21083; 1:500; Abcam, Cambridge, UK) for 1 h at room temperature and washed three times with PBS-T. Subsequently, cells were incubated with Alexa Fluor 647-conjugated goat anti-rabbit IgG secondary antibody (1:500; Invitrogen) for 30 min at room temperature and washed three times with PBS-T. Nuclei were stained with Hoechst 33342 (Thermo Fisher Scientific) according to the manufacturer’s instructions. Images were acquired using a BIOREVO BZ-9000 fluorescence microscope (Keyence, Osaka, Japan). For quantitative analysis, five random fields were imaged, and the number of DSB foci was counted in a total of 70 cells. All data were collected from at least two independent experiments.

### SER and DER

To quantify the degree of enhanced radiosensitivity in cell experiments and to apply these results to the protection of normal cells in clinical practice, we calculated the following experimental SER, which is defined as the ratio of the dose necessary to induce biological effects in the absence of a sensitizer to that required to achieve the same effects with a sensitizer. The experimental SER for survival (SER_SF_) can be calculated by


(1) \begin{equation*} \qquad\qquad\qquad{\mathrm{SER}}_{\mathrm{SF}}=\frac{D_{\mathrm{w}}}{D_{\mathrm{w}\mathrm{o}}} \end{equation*}

where *D*_wo_ is the absorbed dose necessary to induce biological effects for 10% survival in the absence of metal and *D*_w_ is the dose required to achieve the same effect with metal. Similarly, the experimental SER for DSB (SER_DSB_) can be expressed as


(2) \begin{equation*} \qquad\qquad\qquad{\mathrm{SER}}_{\mathrm{DSB}}=\frac{{\mathrm{DSB}}_{\mathrm{w}}}{{\mathrm{DSB}}_{\mathrm{w}\mathrm{o}}} \end{equation*}

where DSB_wo_ is the nuclear 53BP1 foci per Gy in the absence of metal and DSB_w_ is that with metal. The definition in Eq. (2) assumes that the number of 53BP1 foci within 1 h after irradiation is proportional to the absorbed dose in Gy. This assumption is based on the findings that DNA damage yields immediately after irradiation are proportional to the absorbed dose [14] and that 53BP1 foci correspond to DBSs [15].

### Calculation of dose distribution near metals by the PHITS code

To compare with the experimental SER values obtained from *in vitro* experiments, we first calculated the DER by the metal using the PHITS code version 3.29 [16]. The experimental geometry used in this study was reconstructed in the PHITS simulation as closely as possible to the actual experimental settings (see Fig. 1a), considering the incidence of therapeutic X-rays (i.e. 4, 6 and 10 MV) on the cultured cells. The transport of photons and secondary electrons in this geometry was computed using the electron gamma shower (EGS) model [17] implemented in PHITS down to 1 keV. In this calculation, the energy deposited within the cell layer (20 μm thickness) at the bottom of a culture dish was sampled using the [t-deposit] tally, which allows us to obtain the deposition energies in certain regions. This cell layer thickness was chosen based on the previous report (i.e. ~20 μm) [18]. It should be noted that in this simulation, there is no air gap between the cell layer and the metal disc; instead, the dish plastic bottom separates them, which is consistent with the experimental setup. The DER represents the value at the position of the cells in the culture medium, 1 mm away from the metal. This is because the thickness of the plastic bottom of the dish is generally ~1 mm.

Using the deposition energy given by the PHITS code, we calculated the DER, which is defined as the ratio of the energy deposition with metal to that without metal, as shown in Eq. (3), where *E*_wo_ and *E*_w_ represent the energy deposition with and without metal, respectively.


(3) \begin{equation*} \qquad\qquad\qquad\qquad\mathrm{DER}=\frac{E_{\mathrm{w}}}{E_{\mathrm{w}\mathrm{o}}} \end{equation*}

Based on these, we compared the experimental SER, derived from cell survival and DSB yield, with the DER calculated from PHITS. The DER values were calculated with sufficient numbers of particles to make the statistical uncertainty less than a few %.

### Estimation of DSB yields by the PHITS code

In the PHITS code, electron track-structure model (hereafter called PHITS-ETS model) enabling to estimate DNA damage yields is available when the track-structure section is activated within a certain region of the PHITS simulation world [12]. Under exposure to 4–10 MV X-rays, scattered X-rays and electrons are generated at the metal surface and pass through nearby cells. This implies that biological effects can be influenced by these low-energy scattering X-rays and electrons. Using the PHITS-ETS model, we performed a DNA damage simulation to estimate the yield of DSBs.

First, we considered the experimental geometry to measure the surviving fraction and nuclear 53BP1 foci (see Fig. 1). The EGS model [17] was also used to transport electrons in the region where track-structure mode is inactivated. The cutoff energy of electrons for the EGS and PHITS-ETS models was set to be 1 and 7 eV, respectively. Using the spatial patterns of ionizations and electronic excitations, we estimated the DSB yield. Briefly, in the DNA damage estimation model in PHITS, it is assumed that the DSB yield is proportional to the number of linkages (pairs of events within a 10 bp separation) per energy deposition. The detail of the model is summarized in the previous paper [12]. It should be noted that the DSB estimation model has been well verified compared to experimental data and the RBE value was used for evaluating the impact of scattered X-rays generated by DMC in this study [12]. Using the model, we calculated the relative biological effect for DSB (RBE_DSB_) defined as follows:


(4) \begin{equation*} \qquad\qquad\qquad{\mathrm{RBE}}_{\mathrm{DSB}}=\frac{Y_{\mathrm{DSB}^\ast }}{Y_{\mathrm{DSB}\left(\mathrm{ref}\right)}} \end{equation*}

where *Y*_DSB^*^_ and *Y*_DSB(ref)_ are the DSB yield for any irradiation conditions (such as with or without metals) and that after irradiation with 6 MV linac X-rays in the absence of metals, respectively. It should be noted that the RBE value was used for evaluating the impact of scattered X-rays generated by DMC in this study. Using the predicted RBE_DSB_ [Eq. (4)] and the DER [Eq. (2)], SER for DSBs, which is estimated by the PHITS code, SER_EST_, can be expressed by


(5) \begin{equation*} \qquad\qquad\qquad{\mathrm{SER}}_{\mathrm{EST}}=\mathrm{DER}\times{\mathrm{RBE}}_{\mathrm{DSB}} \end{equation*}

It has been known that radiation-induced DSB is intrinsically related to cell death induction. Based on these, comparing the predicted DER and SER_EST_ to the experimental SER (i.e. SER_SF_ or SER_DSB_), we discussed the impacts of low-energy scattering radiations on biological effects. Note that the experimental SER includes the enhancement of biological impacts by changes in absorbed dose and radiation quality, whereas only DER does not reflect the modification of impact by scattering X-rays. The SER_EST_ values were calculated with sufficient numbers of particles to make the statistical uncertainty less than a few %. It is important to note that when the SER value is obtained from biological experiments, it is referred to as the experimental SER, and when it is calculated using PHITS, it is called SER_EST_.

### Preclinical evaluation of dose enhancement

After analyzing the radiosensitization effects of DMC, we also conducted a preclinical evaluation. Two preclinical evaluations were performed to assess the dose enhancement near metals: one using a simple tooth model and the other using a human voxel phantom derived from a Computed Tomography (CT) image. Note that the use of CT-based human phantom is useful for evaluating the radiosensitization effects of DMC in the human body with complex anatomy. In both simulations, the energy deposition was calculated, and the DER value was evaluated based on Eq. (3).

**Fig. 2:**
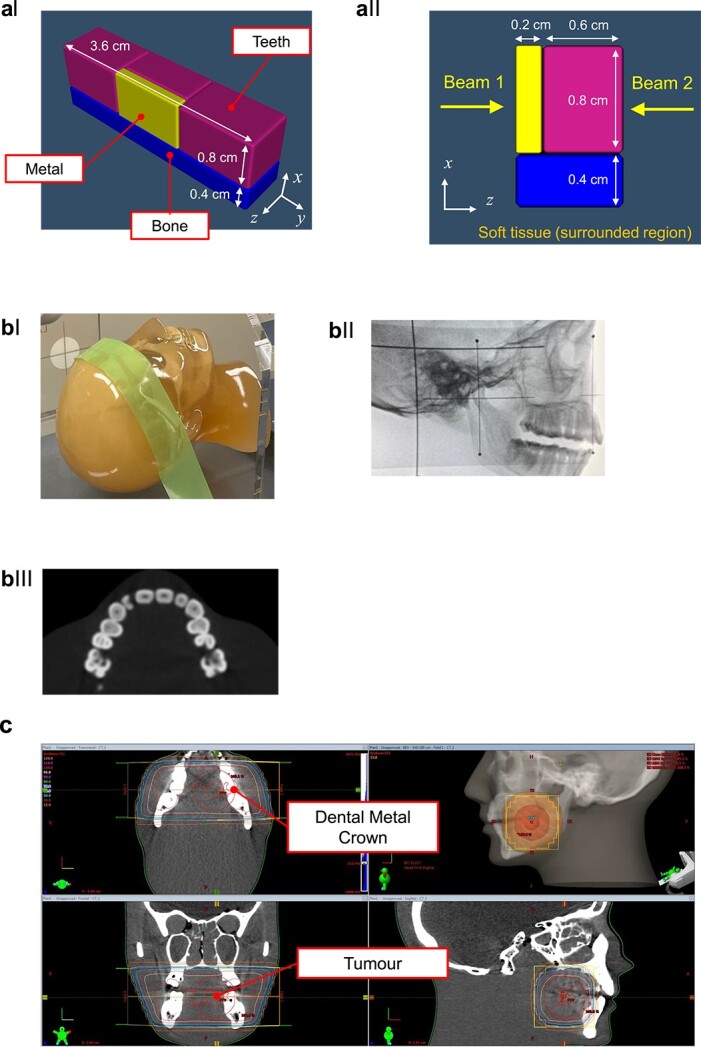
Patient systems, simulations, and phantoms. (a) Simplified numerical phantom model of teeth, metal, and surrounding tissue in the oral cavity, simulated using PHITS. A thin yellow box in the center represents metal, the purple boxes around the upper part represent teeth, the blue box at the bottom represents bone, and gray spaces outside of these structures indicate soft tissue (aI). Yellow arrows extending from both sides show the direction of the beam (aII). (b) The CT phantom (bI), its X-ray image (bII), and its thin-slice CT image (bIII). (c) Structures of a 2 cm-sized tumor and dental metal crown (DMC) in Eclipse. Opposing beams are irradiated from the left and right sides to treat the tongue tumor.

For the simple tooth model, we considered teeth (1.2 × 0.8 × 0.8 cm^3^, 2.75 g/cm^3^), mineral bone (3.6 × 0.4 × 0.4 cm^3^, 1.92 g/cm^3^) and surrounding soft tissue (1.00 g/cm^3^) based on tissue composition reported in ICRU and ICRP110, as shown in Fig. 2a. This model was based on a previous report [4]. 6 MV linac X-rays were incident on the teeth, and the deposition energy was scored using the [t-deposit] tally. Additionally, 2 mm-thick metals, including gold teeth (Au and Pt 98%, Zn 1.8%, others 0.2%), silver teeth (Ag 75%, Zn 10%, Sn 15%) and titanium implant (Ti > 99.43%), were positioned at the surface of the central tooth, as shown in Fig. 2a, and the DER values were calculated.

For the simulation using a human voxel phantom, a CT image of the anthropomorphic head phantom (Kyoto Kagaku, LTD., Kyoto, Japan) was acquired with 0.6 mm-spaced slices (Aqualion, Toshiba, Japan). The phantom is composed of soft tissue equivalent and bone equivalent materials with similar X-ray absorptivity as the human body (see Fig. 2b). The CT image was converted into a human voxel phantom using the Radiotherapy package based on PHITS (RT-PHITS) [19, 20]. A new module of RT-PHITS, Voxel-ID Modifier, was developed to insert additional structures into the created phantom. Using this function, a 2 mm-thick dental metal crown (DMC) was inserted into the nearby first molar of the phantom to study the effect of DMC (Fig. 2a). The metal region was defined by contouring a part of a tooth on the CT image using the Eclipse treatment planning system (Varian Medical Systems, Palo Alto, CA, USA). The Voxel-ID Modifier converted the voxels contained inside the contour into the specified material (Fig. 2b). A treatment plan was created using the Eclipse treatment planning system, assuming a 2 cm-sized metastatic or recurrent tongue tumour on the left lateral margin of the tongue body on the CT image. The tumour was positioned at the isocentre, and the field size was set to 5 × 5 cm. Opposing 6 MV-linac X-ray beams were used for bilateral left–right counter-irradiation from both sides (Fig. 2c). The PHITS simulation was conducted accordingly using the created phantoms with and without the metal region, and the DER values were calculated. In the same manner, the calculations were also calculated with sufficient numbers of particles to make the statistical uncertainty less than a few %.

### Statistical analysis

The statistical analysis was conducted using Pearson’s correlation coefficient test, the Mann–Whitney U test and one-way ANOVA, followed by a *post hoc* Tukey’s multiple comparison test. A *P*-value below 0.05 was considered to indicate statistical significance.

## RESULTS

### Cell survival and the experimental SER values

**Fig. 3:**
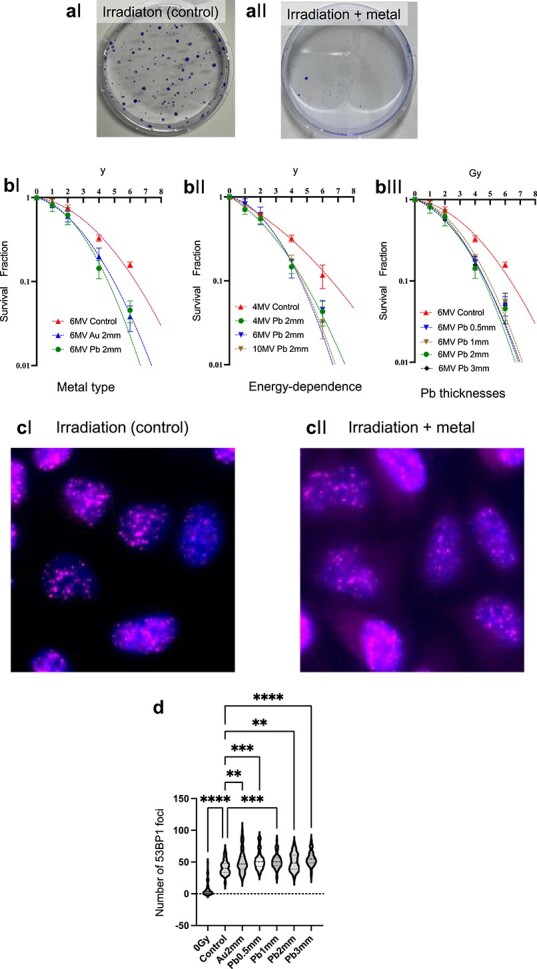
Colony assay results. (a) Appearance of colonies irradiated with 6 MV, 6 Gy X-rays without a metal disc (aI) and with a metal disc (aII). (b) Survival curves comparing control, Au and Pb (bI); different X-ray energies (4, 6 and 10 MV) using a Pb disc (bII); and various thicknesses (0.5–3 mm) of Pb discs (bIII). Compared to their respective controls, the presence of metal discs enhanced radiosensitivity. Using the dose required to induce a 10% survival rate, the experimental SER values were found to be 1.2–1.4. (c) DNA DSB foci induced by 2 Gy irradiation, visualized by immunostaining of 53BP1, without a metal disc (cI) and with a metal disc (cII). Although not immediately apparent, counting the foci within individual nuclei reveals that the presence of metal discs results in a 1.2–1.4-fold increase in the number of foci compared to the control. (d) Violin plot quantifying 53BP1 foci per cell after 2 Gy irradiation, based on data from five randomly chosen fields. Statistical significance is indicated as follows: ^*^^*^ for *P* < 0.01, ^*^^*^^*^ for *P* < 0.001 and ^*^^*^^*^^*^ for *P* < 0.0001.

Figure 3aI and II shows representative images of colonies that were irradiated with and without metal, respectively. Note that Fig. 3aI and II are the representative colony pictures. These mean values obtained by three independent experiments are depicted in Fig. 3bI. As depicted in Fig. 3bI, the survival fraction with metals (Au and Pb) was the lowest among all groups, and no significant difference in radiosensitivity was observed between cells near Au and Pb. Using the dose required to induce a 10% survival rate, the experimental SER value for survival (SER_SF_) was found to be 1.2–1.4. Given the absence of significant differences between Au and Pb, we subsequently investigated the impact of X-ray energy and Pb thickness on the experimental SER values to further understand the factors influencing radiosensitivity.

The radiosensitivity with 2 mm-thick Pb was measured for various X-ray energies, i.e. 4, 6 and 10 MV. The results showed no significant differences between the different X-ray energies (see Fig. 3bII), indicating that the experimental SER value is not strongly dependent on the X-ray energy in the investigated range. Based on these findings, subsequent experiments were carried out using a radiation intensity of 6 MV to maintain consistency and simplify the experimental setup.

Next, we measured the radiosensitivities with Pb for various thicknesses (i.e. 0.5–3 mm) to determine the minimum thickness required to achieve the maximum experimental SER value. As shown in Fig. 3bIII, the results revealed no significant differences in radiosensitivity amongst Pb discs with a thickness of 1 mm or more, suggesting that a thickness of 1 mm is sufficient to achieve the maximum experimental SER value. We also measured the radiosensitivity for different shapes or conditions of Pb (e.g. Pb beads plate and a 2 mm Pb disc with a 1 mm plastic shield) to investigate the influence of metal shape and shielding on the experimental SER values. Although the setup conditions were complex, all factors, including the shape of the metal, the position of the plastic, the position of the metal and their respective thicknesses, were accurately calculated to match the real experimental settings. As a result, the experimental SER with the Pb beads plate was found to be similar to that of the 2 mm Pb. However, reduced radiosensitivity was observed when using a 2 mm Pb disc combined with a 1 mm plastic shield (see Table 1 and Fig. S1), indicating that shielding can reduce the radiosensitization effect. Details of each condition are provided in the supplementary material.

**Table 1:** Experimental SERs at 10% survival for each X-ray energy and condition, determined from the colony assay. ‘Pb distanced’ indicates a 1 mm separation between the Pb disc and the dish using a plastic shield. ‘Pb beads’ refers to the use of 2 mm Pb spheres instead of a Pb disc. ‘Pb shielded’ means that the beam is shielded by placing a 2 mm-thick Pb disc on the lid of the dish

X-ray energy	Conditions	SER	±SD
4 MV	Control	1.00	0.26
4 MV	Pb 2 mm	1.39	0.27
4 MV	Pb 3 mm	1.14	0.27
6 MV	Control	1.00	0.48
6 MV	Pb 0.5 mm	1.35	0.61
6 MV	Pb 1 mm	1.32	0.54
6 MV	Pb 2 mm	1.39	0.84
6 MV	Pb 3 mm	1.37	0.72
6 MV	Au 2 mm	1.38	0.50
6 MV	Pb distanced	1.18	0.52
6 MV	Pb beads	1.23	0.59
6 MV	Pb shielded	0.89	0.38
10 MV	Control	1.00	0.57
10 MV	Pb 2 mm	1.47	0.60
10 MV	Au 2 mm	1.54	0.65
10 MV	Pb distanced	1.25	0.55

### Quantification of nuclear DSBs using 53BP1 focus formation assay

To further investigate the biological effects of metal discs on irradiated cells, we measured the nuclear number of 53BP1 foci, which serve as markers for DSBs. Our observation revealed that the number of these foci increased based on the type of metal and its thickness as depicted in Fig. 3cI and II. Although the differences in 53BP1 foci formation might not be immediately apparent in Fig. 3cI and II, the violin plot in Fig. 3d clearly demonstrates the significant increase in the number of foci in the presence of metal discs compared to the control without metal. Compared to the nuclear number of foci formed after 2 Gy irradiation without metal, the presence of a metal disc significantly increased the number of foci (*P* < 0.003). A violin plot, which quantitatively presents the number of 53BP1 foci, further validated this observation (Fig. 3d). The uncertainties of nuclear foci might due to the heterogeneity of cellular dose and cell-cycle phase [21]. Importantly, our results showed no significant difference in the number of 53BP1 foci between Au and Pb discs of the same thickness. Additionally, we observed no notable difference in DSBs among different thicknesses of Pb. These findings suggest that the quantity and quality of secondary electrons, which arise due to the scattering of X-rays by metal, are comparable regardless of the metal type. This is consistent with the results obtained from the cell survival experiments and further supports the idea that the radiosensitization effect is not strongly dependent on the metal type or thickness, once a minimum thickness is reached.

### Comparison of PHITS simulations with experimental SER

**Fig. 4:**
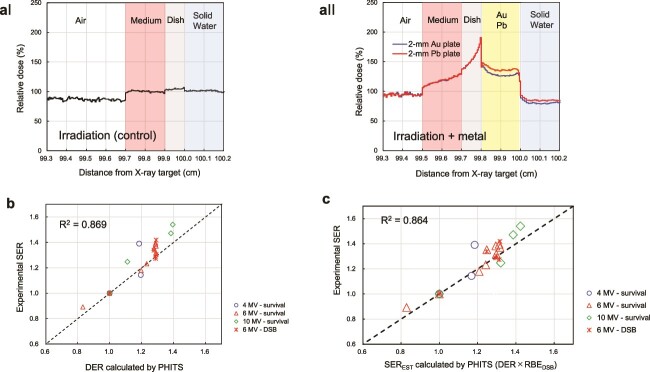
PHITS calculation results. (a) Dose distribution near dishes without metals (aI) and with metals (aII). Regardless of the metal type (Au or Pb), the relative dose at the surface of the dish where the cells are present (*x* = 99.7 [cm]) is 120%–140%. (b) Scatterplot comparing the DER at a distance of 1 mm from the metal, calculated from PHITS, and the experimental SER derived from *in vitro* experiments (survival curves and DSB foci counts). The DER and experimental SER values showed a strong correlation (*R*^2^ = 0.869), indicating that the DER predicted by PHITS is a key factor in determining the experimental SER. (c) Scatterplot comparing the SER_EST_ values calculated by PHITS and the experimental SER values. The SER_EST_ values were obtained by multiplying the DER values from PHITS by the relative biological effectiveness (RBE) for DSB induction. The strong correlation between SER_EST_ and the experimental SER (*R*^2^ = 0.864) suggests that both DSB and SER_EST_ can accurately predict the experimental SER.

To validate the experimental findings and gain insights into the dose enhancement effect of metal discs, we theoretically interpreted the experimental SER values by calculating the dose distribution around the metal using the PHITS code. When the metal is positioned on a water phantom 100 cm from the radiation source, the dose is normalized to that in the cell culture medium without metal (Fig. 4aI and II). In Fig. 4aII, the blue and red lines correspond to the Au and Pb discs, respectively. It is evident that the relative dose, or DER value at the surface of the dish where the cells are present (*x* = 99.7 [cm]) is 120%–140% regardless of the metal type.

To assess the relationship between the experimental SER values and the DER values predicted by the PHITS code, we quantified the ability to form colonies under various conditions and energies (i.e. 4, 6 and 10 MV) and derived the experimental SER at a 10% survival reference point. Additionally, we determined the experimental SER based on the DSB foci count at an X-ray energy of 6 MV. By employing the PHITS code, we calculated the DER and established a scatter plot of the experimental SER versus the DER predicted by PHITS (Fig. 4b). This analysis revealed a strong correlation (*R*^2^ = 0.869), indicating that the PHITS code can accurately predict the dose enhancement effect of metal discs.

Next, we calculated the SER_EST_ by multiplying the DER values with the RBE_DSB_ values obtained from the DSB yield. Figure 4c illustrates the relationship between the SER_EST_ values calculated by PHITS and the corresponding experimental SER values. The strong correlation remained unchanged (*R*^2^ = 0.864) even when considering the SER_EST_ values that account for RBE. In both cases, the predicted DER and SER_EST_ were successful in reproducing the experimental SER, as indicated by the *R*^2^ values.

From the comparisons, the dose increase is a key factor in explaining the enhanced radiosensitivities of clonogenic cell survival and initial DSB induction. This strong correlation validates the use of the PHITS code for simulating the dose enhancement effect of metal discs in a clinical setting. This finding is crucial for the development of accurate treatment planning systems that can account for the presence of metal implants or dental materials in patients undergoing radiotherapy.

### Simulation of palliative irradiation of metastatic or recurrent tongue tumours

**Fig. 5:**
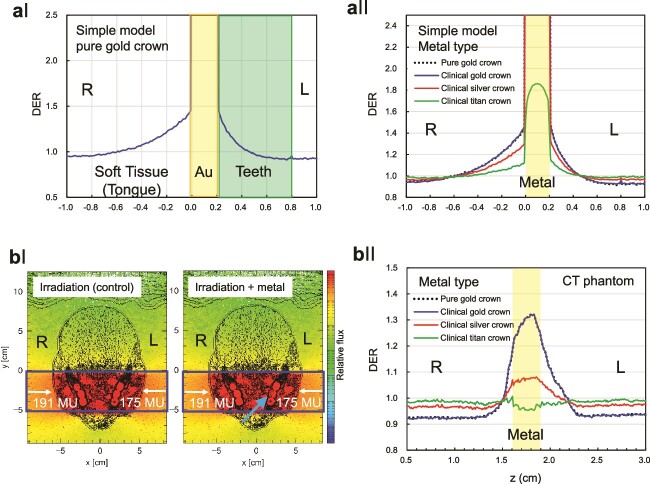
Simulation results using phantoms in PHITS. (a) Coronal section dose distribution in a simple tooth model with a pure gold crown (aI). Comparison of dose distributions for different metals in the simple tooth model. Black dotted, blue, red and green lines show the results for pure gold, gold crown, silver crown and titanium, respectively (aII). The lines for pure gold and gold crown overlap, indicating nearly identical values. The titanium line is lower than the others, showing a reduced dose compared to other metals. (b) Axial cross-sectional dose distribution in the voxel-based CT phantom. ‘L’ and ‘R’ are indicated to explicitly show the left–right orientation. Blue arrows indicate the contours of the curved crown (bI). Comparison of dose distributions for different metals in the voxel-based CT phantom. Light transparent yellow areas represent the estimated regions of the crowns, comparing the differences among various metals (bII).

Having established the accuracy of the PHITS code in predicting the dose enhancement effects of metal discs, we applied our findings to a real-life clinical scenario to demonstrate the potential impact of DMCs on the dose distribution in the oral cavity during radiotherapy. We chose the case of palliative irradiation for metastatic or recurrent tongue cancer as an example, as it represents a common clinical situation where the presence of DMCs may influence the treatment outcome. We simulated two opposed beam irradiations to the oral cavity using the PHITS code and evaluated the DER distribution around the DMC. In Fig. 5, ‘L’ and ‘R’ are indicated to explicitly show the left–right orientation, providing a precise understanding of the spatial relationship between the irradiation beams and the oral cavity structures.

First, the DER distribution around DMC was calculated using a simple tooth model. Figure 5aI shows the coronal section dose distribution in the simple tooth model. In this model, the DER values for both the soft tissue (assumed to be the tongue) and the teeth nearest to the Au were found to be 1.43. As with pure gold, we calculated dose distributions for different metals used in dental materials, comparing their effects (Fig. 5aII). For both the pure and clinical gold crown, the maximal DER to the tongue was 1.44 and to the tooth 1.42; as in the *in vitro* experiment, the DER value at 1 mm away from the metal was 1.33 on the tongue side and 1.2 on the tooth side. For silver crowns, the maximal DER values for the tongue and the tooth were 1.28 and 1.29, respectively. For titanium crowns, the maximal DER values for the tongue and the teeth were 1.11 and 1.13, respectively. All the calculated values were within the range of the graph and did not exceed the frame.

Next, the calculation results are shown in a voxel model using the head phantom CT data with very fine slices of 0.6 mm spacing. Figure 5bI shows the axial cross-sectional view of the fluence map, which represents the number of particles per unit area integrated over the irradiation time. The radiation beams were irradiated from both the right (191 MU) and the left (175 MU) sides of the phantom. The rainbow colours indicate that the redder area received higher photon fluence. The left side of the figure corresponds to the patient’s right. Figure 5bII shows the calculation results of DER in the presence of metal. It is important to note that the results are for reference only, as the boundaries of the structures cannot be identified precisely due to the limitations of the voxel model. The curved surface of the gold crown and the characteristics of the voxel model make it challenging to determine the exact boundary points of the metal surface. As a result, the indicated boundary positions in the dose distribution, i.e. ~1.7 and 1.9 cm in the *z*-direction, should be understood as estimated approximations and do not precisely represent the metal surface (see Fig. 5bII). These limitations should be considered when interpreting the results obtained from the voxel model.

As shown in Fig. 5bII, the DERs for both the pure and clinical gold crown were evaluated at positions ~1.7 and 1.9 cm in the *z*-direction for reference. The soft tissue on the right side of the figure is assumed to be the tongue. The maximal DER value was 1.27, observed in both the tongue and teeth. It should be noted that these values at the approximate positions in the *z*-axis are for reference only. The obtained DER values were lower than those derived from the simple tooth model, a trend also observed for silver and titanium teeth. The differences in DER values between the simple tooth model and the voxel model can be attributed to the more realistic representation of the human anatomy in the voxel model, which accounts for the complex geometry and heterogeneous composition of the oral cavity. The lower DER values obtained from the voxel model suggest that the dose enhancement effect of DMCs may be less pronounced in a clinical setting compared to the idealized conditions of the simple tooth model. However, the voxel model still demonstrates the potential impact of DMCs on the dose distribution in the oral cavity during radiotherapy, highlighting the need for accurate treatment planning systems that can account for the presence of dental materials.

## DISCUSSIONS

In this study, we employed several experimental approaches to investigate the effects of metal presence on radiation dose and subsequent cellular damage. We placed a metal disc under a culture dish and irradiated it with X-rays ranging from 4 to 10 MV. Additionally, we used the DICOM data, obtained from CT scans of a phantom designed to mimic human tissue, in simulations run using the PHITS code. The DER, which correlates with SER_EST_, was also calculated to account for the impacts of low-energy secondary electrons released from the metal. To quantitatively evaluate the impacts, we also performed DNA damage simulation in the presence of metals in the PHITS calculation and obtained a strong correlation between experimental SER and the estimated DER.

*In vivo*, the experimental SER might be higher at a distance equivalent to the diameter of a single cell (e.g. ~10–30 μm) from the metal, indicating a potentially significant biological effect. However, in our *in vitro* setup, cells cannot be cultured directly on the metal, making it challenging to measure and evaluate the direct effects of the metal on radiation dose enhancement at such a short distance. This limitation in our *in vitro* model suggests that our findings may underestimate the radiosensitization effects observed in the clinical setting, highlighting the need for an innovative method to replicate and study this phenomenon more accurately. Additionally, a limitation that should be considered is the slight difference between the actual experiment and the PHITS simulation, which indicates the necessity of further theoretical evaluation. However, considering the good agreement between the simulation results and the corresponding experimental data, the difference seems to be negligible.

Another notable finding in this study was that no change in both experimental SER and SER_EST_ was observed even when the metal was thicker than 0.5 mm. This indicates that metals with heavier atomic numbers scatter more secondary electrons, even if they are thinner metal discs. Furthermore, the PHITS code can accurately simulate the SER_EST_ at any metal thickness. Although we only tested Pb and pure Au in this study, similar experiments could be performed with other metals, such as other dental metals. However, even for Pb and Au with high atomic numbers, the experimental SER is ~1.20. For metals with low atomic numbers like titanium, the experimental SER is below 1.10, which might not be significant.

*In silico*, we calculated the relative dose increase to the mucosa around DMCs using two different models: a simplified numerical model of the oral cavity and a voxel model derived from head CT scans. Although the maximum DER observed in the simplified numerical model was 1.43, in agreement with previous studies [4, 5], the DER value decreases to 1.20 in the voxel model derived from head CT scans. Due to the complex human structure, precisely locating the point of maximal DER can be challenging. This is because determining the border between metal and human tissue might be difficult when calculating the value using over a finite-sized volume in the model at the mm-spatial resolution. To deepen our understanding of these effects and improve the accuracy of these simulations, further studies employing numerical phantoms that more closely replicate the detailed anatomy of the human body are necessary. In clinical settings, the most accurate approach to calculate the dose distribution would be to create personalized phantoms based on each patient’s unique anatomy. However, generating such individualized phantoms for every patient undergoing radiotherapy poses significant practical difficulties and may not be feasible due to the time, resources and expertise required. Implementing patient-specific phantoms on a routine basis would necessitate a streamlined workflow, advanced imaging techniques and specialized software tools, which could be challenging to integrate into existing clinical protocols.

The present work shows that it is possible to accurately reproduce the amounts of secondary electrons emitted from DMCs present in the oral cavity during radiotherapy. This may increase the risk of high-dose exposure to the mucosa and mucositis. The model case used in this study was constructed based on a real-world scenario of palliative irradiation of 10 fractions of 30 Gy to a patient with metastatic and recurrent tongue cancer. It is generally accepted that there is a Grade 2–3 threshold at a dose of 43.0 Gy to the oral mucosa [22] and that a total dose of 39.1 Gy or more produces mucositis lasting >3 weeks [23]. If the DER at a 1 mm distance from DMCs is between 1.1 and 1.4, this equates to an irradiation dose of 33–42 Gy, administered in 10 fractions. Assuming an α/β ratio of 10 Gy for the oral mucosa, this means that 37–50 Gy in equivalent dose in 2 Gy fractions (EQD2) would have been irradiated. It could be explained by the fact that only the mucosa around the DMC was localized and exceeded the dose threshold, resulting in localized and intense mucositis.

Our results demonstrate that DER diminishes sharply as the distance from the DMC increases. This observation suggests that mucositis, a common side effect of radiotherapy, could be prevented by maintaining a separation between the oral mucosa and the DMC. A simple and practical approach to achieve this separation is by placing moist gauze or cotton balls between the mucosa and the DMC during treatment. This method could potentially replace the need for specialized oral stents [24, 25], which can be uncomfortable for patients and require additional resources to fabricate. Furthermore, the PHITS code could be employed to accurately calculate DERs in these scenarios, providing valuable insights for treatment planning. The development of advanced, high-resolution human numerical phantoms, which offer a more realistic representation of anatomy compared to traditional voxel phantoms, could pave the way for highly precise treatment planning in head and neck cancer radiotherapy. These next-generation phantoms would enable more accurate submillimetre dose assessments and better account for the presence of dental materials, ultimately leading to improved treatment outcomes and reduced side effects for patients.

## CONCLUSIONS

We conducted a hybrid study with cell experiments and MC simulations (the PHITS code) to investigate the radiosensitizing effects near oral metallic materials. The *in vitro* experiments showed SER values consistent with the dose increase calculated using the PHITS code, highlighting that the radiosensitizing effects near metal are predominantly attributed to the dose increase.

However, CT-based dose assessments using MC simulations underestimated DER values due to complex human anatomy and limited spatial resolution. The development of high-resolution individual human phantoms is crucial for precise dose calculations around dental metallic components, which would be expected to improve treatment planning for head and neck cancer patients.

## ACKNOWLEDGEMENTS

The authors express their gratitude to Medikit Co. Ltd. for providing the gold and assistance in processing the Au disc. The authors also thank all the radiation therapy staff members for their support in irradiating the dishes using the radiation therapy machine and Kenta Suzuki from Soka Municipal Hospital for his assistance in the modeling of PHITS.

## CONFLICT OF INTEREST

The authors declare that they have no competing interests.

## FUNDING

This work was supported by JSPS KAKENHI Grant Number JP22H03744, JP21K15779, JP23K14889.

## DATA AVAILABILITY

All datasets generated and analyzed during this study are freely available and can be obtained by contacting the corresponding author or are included within the article and its supplementary files.

## Supplementary Material

Supplementary_Clean_rrae062

## References

[1] Joiner MC, van der Kogel AJ, Steel GG. Introduction: The Significance of Radiobiology and Radiotherapy for Cancer Treatment. In: Joiner M, van der Kogel A (eds). Basic Clinical Radiobiology, Fourth edn. London: Hodder Arnold Publication, 2009, 1–10. 10.1201/b13224-2.

[2] Sugano Y, Mizuta M, Takao S et al. Optimization of the fractionated irradiation scheme considering physical doses to tumor and organ at risk based on dose-volume histograms. Med Phys 2015;42:6203–10. 10.1118/1.4931969. 26520713

[3] Marta GN, Silva V, de Andrade CH et al. Intensity-modulated radiation therapy for head and neck cancer: systematic review and meta-analysis. Radiother Oncol 2014;110:9–15. 10.1016/j.radonc.2013.11.010. 24332675

[4] Chin DWH, Treister N, Friedland B et al. Effect of dental restorations and prostheses on radiotherapy dose distribution: a Monte Carlo study. J Appl Clin Med Phys 2009;10:80–9. 10.1120/jacmp.v10i1.2853. 19223833 PMC5720502

[5] Kamomae T, Itoh Y, Okudaira K et al. Dosimetric impact of dental metallic crown on intensity-modulated radiotherapy and volumetric-modulated arc therapy for head and neck cancer. J Appl Clin Med Phys 2016;17:234–45. 10.1120/jacmp.v17i1.5870. 26894359 PMC5690192

[6] Mian TA, Van Putten MC, Jr KDC et al. Backscatter radiation at bone-titanium interface from high-energy X and gamma rays. Int J Radiat Oncol Biol Phys 1987;13:1943–7. 10.1016/0360-3016(87)90364-6. 3679935

[7] Wang R, Boyle A. A convenient method for guarding against localized mucositis during radiation therapy. J Prosthodont 1994;3:198–201. 10.1111/j.1532-849X.1994.tb00155.x. 7866501

[8] Reitemeier B, Reitemeier G, Schmidt A et al. Evaluation of a device for attenuation of electron release from dental restorations in a therapeutic radiation field. J Prosthet Dent 2002;87:323–7. 10.1067/mpr.2002.122506. 11941360

[9] Sicard-Roselli C, Brun E, Gilles M et al. A new mechanism for hydroxyl radical production in irradiated nanoparticle solutions. Small 2014;10:3338–46. 10.1002/smll.201400110. 24863679

[10] Matsuya Y, Ohtsubo Y, Tsutsumi K et al. Quantitative estimation of DNA damage by photon irradiation based on the microdosimetric-kinetic model. J Radiat Res 2014;55:484–93. 10.1093/jrr/rrt222 24515253 PMC4014172

[11] Yachi Y, Yoshii Y, Matsuya Y et al. Track structure study for energy dependency of electrons and X-rays on DNA double-strand break induction. Sci Rep 2019;9:17649. 10.1038/s41598-019-54081-6. 31776470 PMC6881292

[12] Matsuya Y, Kai T, Sato T et al. Track-structure modes in particle and heavy ion transport code system (PHITS): application to radiobiological research. Int J Radiat Biol 2022;98:148–57. 10.1080/09553002.2022.2013572 34930091

[13] Friedrich T, Pfuhl T, Scholz M. Update of the particle irradiation data ensemble (PIDE) for cell survival. J Radiat Res 2021;62:645–55. 10.1093/jrr/rrab034. 33912970 PMC8273790

[14] Ljungman M, Nyberg S, Nygren J et al. DNA-bound proteins contribute much more than soluble intracellular compounds to the intrinsic protection against radiation-induced DNA strand breaks in human cells. Radiat Res 1991;127:171–6. 10.2307/3577962. 1947001

[15] Ward IM, Minn K, Jorda KG, Chen J. Accumulation of checkpoint protein 53BP1 at DNA breaks involves its binding to phosphorylated histone H2AX. J Biol Chem 2003;278:19579–82. 10.1074/jbc.C300117200. 12697768

[16] Sato T, Iwamoto Y, Hashimoto S et al. Features of particle and heavy ion transport code system (PHITS) version 3.02. J Nucl Sci Technol 2018;55:684–90. 10.1080/00223131.2017.1419890.

[17] Hirayama H, Namito Y, Bielajew AF, Wilderman SJ, Nelson WR. The EGS5 Code System. SLAC-R-730 and KEK Report 2005-8. Stanford, CA: Stanford Linear Accelerator Center; Tsukuba, Japan: High Energy Accelerator Research Organization; 2005.

[18] Qiu X, Lombardo JA, Westerhof TM et al. Microfluidic filter device with nylon mesh membranes efficiently dissociates cell aggregates and digested tissue into single cells. Lab Chip 2018;18:2776–86. 10.1039/c8lc00507a. 30090895 PMC6171522

[19] Furuta T, Koba Y, Hashimoto S et al. Development of the DICOM-based Monte Carlo dose reconstruction system for a retrospective study on the secondary cancer risk in carbon ion radiotherapy. Phys Med Biol 2022;67:145002. 10.1088/1361-6560/ac7998. 35781266

[20] Sato T, Furuta T, Liu Y et al. Individual dosimetry system for targeted alpha therapy based on PHITS coupled with microdosimetric kinetic model. EJNMMI Phys 2021;8:4. 10.1186/s40658-020-00350-7. 33432383 PMC7801536

[21] Mori R, Matsuya Y, Yoshii Y, Date H. Estimation of the radiation-induced DNA double-strand breaks number by considering cell cycle and absorbed dose per cell nucleus. J Radiat Res 2018;59:253–60. 10.1093/jrr/rrx097. 29800455 PMC5967466

[22] Musha A, Shimada H, Shirai K et al. Prediction of acute radiation Mucositis using an oral mucosal dose surface model in carbon ion radiotherapy for head and neck Tumors. PLoS One 2015;10:e0141734. 10.1371/journal.pone.0141734. 26512725 PMC4626117

[23] Narayan S, Lehmann J, Coleman MA et al. Prospective evaluation to establish a dose response for clinical oral mucositis in patients undergoing head-and-neck conformal radiotherapy. Int J Radiat Oncol Biol Phys 2008;72:756–762.e4. 10.1016/j.ijrobp.2008.01.060. 18417299

[24] Kaanders JH, Fleming TJ, Ang KK et al. Devices valuable in head and neck radiotherapy. Int J Radiat Oncol Biol Phys 1992;23:639–45. 10.1016/0360-3016(92)90023-b. 1612965

[25] Ben-David MA, Diamante M, Radawski JD et al. Lack of osteoradionecrosis of the mandible after intensity-modulated radiotherapy for head and neck cancer: likely contributions of both dental care and improved dose distributions. Int J Radiat Oncol Biol Phys 2007;68:396–402. 10.1016/j.ijrobp.2006.11.059. 17321069 PMC2702207

