# Supplementary material for: In vitro and in silico study of biological effects on cancer cells in the presence of metallic materials during radiotherapy
Source: J Radiat Res. 2024 Aug 22;65(5):628–39. doi: 10.1093/jrr/rrae062 (PMC11420842; doi:10.1093/jrr/rrae062)
Supplement: Supplementary_Clean_rrae062 [file supplementary_clean_rrae062.docx]

Supplementary

***Colony Formation Assay Under Various Conditions***

Colony formation assays were performed using different configurations, including Au and Pb discs, as well as other setups. In some assays, 2 mm diameter Pb spheres (Hands Corporation, Tokyo, Japan) were placed on a flat surface, and the culture dish was positioned on top of them (Fig. S1A-C). These Pb spheres were used to mimic the effect of Au spheres, which are occasionally implanted in the body as fiducial markers, and to investigate the impact of shape on the results. In other assays, the culture dish was either shielded with a Pb disc or separated from the Pb disc by a 1 mm-thick acrylic sheet (Acrysunday Co., Ltd. Tokyo, Japan), effectively increasing the distance between the disc and the cells from 1 mm to 2 mm (Fig. S1D). These experimental conditions were also simulated using the PHITS code. Although detailed data are not presented here, the results of these experiments are represented by the data points in Fig. 4b and Fig. 4c.

Fig. S1. Overview of the experimental setups. (A) Dishes on each metal disc. (B) A 1-mm thick Pb disc. (C) Two mm diameter Pb spheres, laid flat on the dish lid. (D) Plastic separators keeping the metal disc from touching the bottom of the dish.
